# Detecting soil-transmitted helminth and *Schistosoma mansoni* eggs in Kato-Katz stool smear microscopy images: A comprehensive in- and out-of-distribution evaluation of YOLOv7 variants

**DOI:** 10.1371/journal.pntd.0013234

**Published:** 2025-07-03

**Authors:** Mohammed Aliy Mohammed, Esla Timothy Anzaku, Peter Kenneth Ward, Bruno Levecke, Janarthanan Krishnamoorthy, Wesley De Neve, Sofie Van Hoecke

**Affiliations:** 1 IDLab, Ghent University - imec, Ghent University, Ghent, Belgium; 2 School of Biomedical Engineering, Jimma Institute of Technology, Jimma University, Jimma, Ethiopia; 3 Department of Electronics and Information Systems, Ghent University, Ghent, Belgium; 4 Center for Biosystems and Biotech Data Science, Ghent University Global Campus, Incheon, South Korea; 5 Enaiblers AB, Uppsala, Sweden; 6 Department of Translational Physiology, Infectiology and Public Health, Ghent University, Merelbeke, Belgium; Stony Brook University, UNITED STATES OF AMERICA

## Abstract

**Background:** Soil-transmitted helminth (STH) and *Schistosoma mansoni* (*S. mansoni*) infections remain significant public health concerns in tropical and subtropical regions. Deep Convolutional Neural Networks (DCNNs) have already shown promising accuracy in identifying STH and *S. mansoni eggs* in the same, in-distribution (ID) settings. However, their performance in real-world, out-of-distribution (OOD) scenarios, characterized by variations in image capture devices and the appearance of previously unseen egg types, has not been thoroughly investigated. Assessing the robustness of DCNNs under these challenging conditions is crucial for ensuring their reliability in field diagnostics.

**Methodology:** Our study addresses the gap in evaluating DCNNs for identifying STH and *S. mansoni* eggs by rigorously testing multiple variants of the You Only Look Once (YOLO) version 7 model under two OOD conditions: (*i*) a dataset shift due to a change in the image capture device, and (*ii*) a combination of this device change and the presence of two egg types not occurring during training. We adopted a 2 × 3 montage data augmentation strategy to enhance OOD generalization. Additionally, we used the Toolkit for Identifying object Detection Errors (TIDE) and Gradient-weighted Class Activation Mapping (Grad-CAM) to perform a comprehensive analysis of the results.

**Principal findings:** In ID settings, YOLOv7-E6E outperformed other models, achieving an F1-score of 97.47%. For the OOD scenario involving only a change in the image capture device, the 2 × 3 montage strategy significantly enhanced performance, increasing precision by 8%, recall by 14.85%, and *mAP*@*IoU*_0.5_ by 21.36%. However, for the more complex OOD scenario that involves both a change in the capture device and the introduction of two previously unseen egg types, the proposed augmentation technique, while beneficial, did not fully address the generalization challenges across all YOLOv7 variants, highlighting the necessity of testing beyond ID scenarios, on which state-of-the-art models predominantly focus.

**Conclusions/significance:** This study underscores the critical importance of utilizing comprehensive test sets and conducting rigorous OOD evaluations when designing machine learning solutions for STH, *S. mansoni* or any other helminth infections. Understanding the true capabilities of DCNNs in real-world settings depends on such thorough testing. Expanding AI-driven diagnostic assessments to account for the complexities encountered in the field is essential for creating robust tools that can significantly contribute to the global elimination of STH and *S. mansoni* infections as public health problems by 2030, a goal put forth by the World Health Organization.

## Author summary

We evaluated the effectiveness of YOLOv7 and its variants—deep learning-based computer vision models designed for object detection—in identifying four types of parasite eggs in stool smears. These models, even the simpler variants, performed exceptionally well in the same, in-distribution (ID) settings, indicating a potential for use by in-field edge diagnostic devices, provided that both the development and real-world deployment scenarios are exactly the same. Nevertheless, in practice, we frequently encounter a multitude of different scenarios that previous parasite egg detection models have not adequately addressed. Our experiments and findings highlight the critical need for thorough testing of out-of-distribution (OOD) scenarios to evaluate the effects of distribution shifts, including changes in capturing devices and the encounter of previously unseen egg classes. This OOD testing is essential to ensure the resilience of models in confronting the unpredictable conditions they may face in real-world scenarios. Such testing is crucial for realizing a trustworthy AI-powered in-field diagnostic device that can help eliminate Soil-transmitted helminth (STH) and *Schistosoma mansoni* (*S. mansoni*) infections as public health problems by 2030.

## 1. Introduction

Soil-transmitted helminth (STH) and *Schistosoma mansoni* (*S. mansoni*) infections continue to pose significant public health challenges, especially in tropical and subtropical regions. These often-overlooked parasitic worm infections, impacting over a billion individuals globally, contribute substantially to the global disease burden, particularly in marginalized communities in sub-Saharan countries where basic healthcare, safe drinking water, and sanitation are lacking [1]. The World Health Organization (WHO) reports that in 2021 alone, STH infections were responsible for 1.38 million disability-adjusted life-years (DALYs), and intestinal schistosomiasis infections, predominantly caused by *S. mansoni*, accounted for 1.75 million DALYs, reflecting the extensive human cost in terms of sickness, disability, or early death [2].

Historically, diagnosing STH and *S. mansoni* infections has depended largely on microscopic examination of stool smears [3–5]. Specifically, the Kato-Katz (KK) technique is widely used to inform large-scale deworming programs targeting STH and *S. mansoni* infections. The technique involves collecting a small stool sample, preparing a smear on a microscope slide, and treating it with a special solution to make parasite eggs easier to see. After a short waiting period, the sample is examined under a microscope, and the number of eggs is counted to estimate the intensity of infection. The results are then interpreted and classified based on WHO-recommended thresholds to determine infection severity [6]. While this technique is often inexpensive and straightforward, the examination of the slides is time consuming (80 percent of the total time to result) [7] and requires substantial expertise, and is marked by variable sensitivity and specificity [8]. Additionally, certain infections, such as hookworm, are challenging to detect due to the rapid degradation of their eggs [9]. Recently, a proof-of-concept artificial intelligence-based digital pathology (AI-DP) system, was developed to automate the image acquisition and analysis of KK smears. The AI-DP integrates multiple subsystems, enabling electronic data capture, whole-slide imaging (WSI), AI-based parasite egg detection, AI-assisted result verification, and cloud-based reporting and monitoring [5,10,11]. In the short term, the AI-DP system is intended to support trained healthcare personnel in mass screening and monitoring programs aligned with the WHO’s road map for neglected tropical diseases [1], aiming to mitigate human-induced errors and reduce the processing time associated with conventional diagnostic methods. To contribute to this road map, this study specifically focuses on the parasite detection subsystem, with the goal of realizing a trustworthy AI model.

The advent of AI in recent years has signaled a potential paradigm shift in automating the diagnostic process [12–21]. Most notably, Deep Conventional Neural Networks (DCNNs) have demonstrated remarkable effectiveness in analyzing microscopy stool smear images. Exhibiting high effectiveness in the same, in-distribution (ID) settings, DCNNs have shown remarkable ability in identifying and differentiating between STH and *S. mansoni* eggs [10]. This progress brings us closer to a future where diagnostics are not only automated but also rapid and reliable, resonating with the evolving landscape of digital pathology.

Despite these advancements, the practical deployment of DCNNs in real-world field diagnostics presents unique challenges, especially under conditions different from those seen during training (out-of-distribution, OOD). In contrast, most state-of-the-art (SOTA) models are evaluated on familiar scenarios that closely match their training data (ID). In OOD settings, such as variations in image capture devices and the encounter with previously unseen egg types can considerably degrade model performance. Therefore, a thorough assessment of DCNN robustness under these OOD conditions is critical to ensure their reliability in diverse diagnostic environments.

To assess the robustness of DCNNs, our study performs a comprehensive evaluation of the You Only Look Once (YOLO) version 7 model, a SOTA framework for real-time object detection, within the specific context of detection of STH and *S. mansoni* eggs. Our objective is to help fill the existing void in DCNN evaluation by in-depth testing of various YOLOv7 variants under two distinct OOD conditions: a shift in the dataset due to changes in image capture devices, and a compounded challenge of device change coupled with the introduction of two egg types not seen during the training phase. To strengthen the adaptability of the models to these demanding conditions, we also present a novel 2 × 3 montage data augmentation strategy. Finally, our approach also includes the use of state-of-the-art tools for investigating error dynamics and model performance, namely the toolkit for identifying object detection errors (TIDE) and Gradient-weighted Class Activation Mapping (Grad-CAM).

Our study represents an important step towards realizing the potential of AI-driven tools in enhancing diagnostic accuracy for STH and *S. mansoni* infections on top of the technical validation of DCNNs for parasite egg detection. As such, this effort aligns with the ‘Ending the Neglect to Attain Sustainable Development Goals’ initiative of the WHO, which targets the elimination of 20 neglected tropical diseases, including STH and *S. mansoni* infections, as public health problems by 2030. The presented study, therefore, carries significant implications for the future of parasitology, public health strategy, and the broader field of medical diagnostics.

In summary, the contributions of our study to the field of parasite egg detection are threefold:

- **Dataset refinement:** We enhanced the AI4NTD KK2.0 P1.5 STH & SCHm Dataset, as introduced in [10], by rectifying intrinsic annotation errors. This led to the development of an improved dataset version (AI4NTD KK2.0 P1.5 STH & SCHm Dataset V2), resulting in more effective models.
- **Model robustness and generalizability:** Our investigation into the robustness of models under OOD conditions demonstrated the need for OOD evaluation. We also showed that a simple, prior knowledge-guided data augmentation technique is able to substantially improve model effectiveness in diverse data environments.
- **Analytical insights:** Through analysis using the **T**oolkit for **I**dentifying **O**bject **D**etection **E**rrors (TIDE), we identified specific causes for the drop in Average Precision (AP) of our models, particularly highlighting localization and classification errors. Additionally, the application of **Grad**ient-weighted **C**lass **A**ctivation **M**aps (Grad-CAM) offered valuable insights into the decision-making process of our models, especially in explaining instances of false positives (FP) and false negatives (FN).

## 2. Related work

Several recent studies have employed DCNN-based object detectors to identify parasite eggs in stool samples. For instance, Qiaoliang *et al*. [17] developed ‘FecalNet’, an object detector with ResNet152 as its backbone, to identify six components in human stool samples and achieved a mAP of 92.16%. Among these identified components were hookworm, *Ascaris lumbricoides (A. lumbricoides)*, and *Trichuris trichiura (T. trichiura)*. Ariel *et al*. [18] designed a smartphone system, ‘KanKanet’, that employs MobileNet for the detection of STH eggs in fecal samples, including *A. lumbricoides*, *T. trichiura*, and hookworm. The system achieved a sensitivity of 82.9% and a specificity of 97.1%. Similarly, Dacal *et al*. [21] used MobileNet to design an automatic detection and quantification system for *T. trichina*, tailored to telemedicine applications with a precision of 98.44±2, a recall of 80.94±9.96, and an F1 score of 88.5±5.73. Ruiz-Santaquiteria *et al*. [19] utilized the widely known DCNN-based two-stage object detector, Faster R-CNN, to identify *S. mansoni* eggs and attained an average precision (AP) of 76.5%. Next, they also created an ensemble of five object detection models to detect eleven types of parasitic eggs in faecal smear samples [20]: (1) Faster-RCNN, (2) one-stage object detection (TOOD), (3) Casc M-RCNN with a Swin-Transformer backbone, (4) Casc M-RCNN with a ConvNeXt-Large backbone, and (5) YOLOX. Among the eleven types of parasitic eggs were *A. lumbricoides* , *T. trichiura*, and hookworm. The ensemble achieved an F1-score of 97.4%.

Despite demonstrating high effectiveness on ID test settings, these studies have not showcased their robustness under OOD conditions. This raises questions about the effectiveness of the models in real-world clinical deployment, which often involves diverse and variable sample preparations, as well as different image acquisition and presentation methods, such as dedicated in-field devices [10], microscope-mounted cameras, and smartphones [22]. Consequently, the reliability of these models against data that deviates from their training set is an area of uncertainty and warrants further investigation.

## 3. Materials and methods

### 3.1. Ethics statement

Our research utilized two distinct datasets: the AI4NTD KK2.0 P1.5 STH & SCHm dataset (hereafter referred to as the P1.5 dataset) and the AI4NTD KK2.0 P3.0 STH & SCHm dataset (hereafter referred to as the P3.0 dataset). Each dataset had specific ethical considerations.

For the P1.5 dataset, no new data collection was performed. This publicly available dataset [10] had all ethical considerations addressed by its original authors. Ethical approvals for the studies contributing to the P1.5 dataset were obtained from the institutional review board of the University Hospital and Faculty of Medicine and Health Sciences, Ghent University, Belgium, as well as local ethics committees in Cambodia (EC2018/1038, Ministry of Health of Cambodia (NEHCR 101)), Ethiopia (EC2019/1289, Arba Minch University), Tanzania (EC2018/1141, Zanzibar Health Research Institute (ZAHREC/03/DEC/2018)), and Kenya (NTD-SC/NCT #:158, Safe Water and AIDS Project (SWAP), Kisumu). For studies involving children, informed consent was obtained from their parents or guardians. No personal participant data were stored in the dataset.

For the P3.0 dataset, data collection adhered strictly to ethical guidelines and was conducted in collaboration with local institutions. The study in Ethiopia, titled “Evaluation of an Artificial Intelligence Driven Digital Platform against Target Product Profiles for Soil-Transmitted Helminthiasis and Schistosomiasis Control Programs,” was reviewed and approved by the Institutional Review Board of Jimma University Institute of Health (Approval number: JHRPGD/555/21) on 31st May 2021. In Uganda, the study was part of the “Integrated Community-based Survey for Program Monitoring” for soil-transmitted helminthiasis and schistosomiasis precision mapping and was reviewed and approved by the Research Ethics Committee at the Vector Control Division, Ministry of Health (Approval number: VCDREC154) on 18th November 2021. Written informed consent was obtained from all adult participants. For minors, consent was provided by their parents, accompanied by the verbal assent of the children. Participant confidentiality was maintained through pseudo-anonymised identifiers. Animal samples included in the P3.0 dataset, collected from Antwerp Zoo, did not require ethical approval as they were obtained during the cleansing of enclosures.

**Fig 1:**
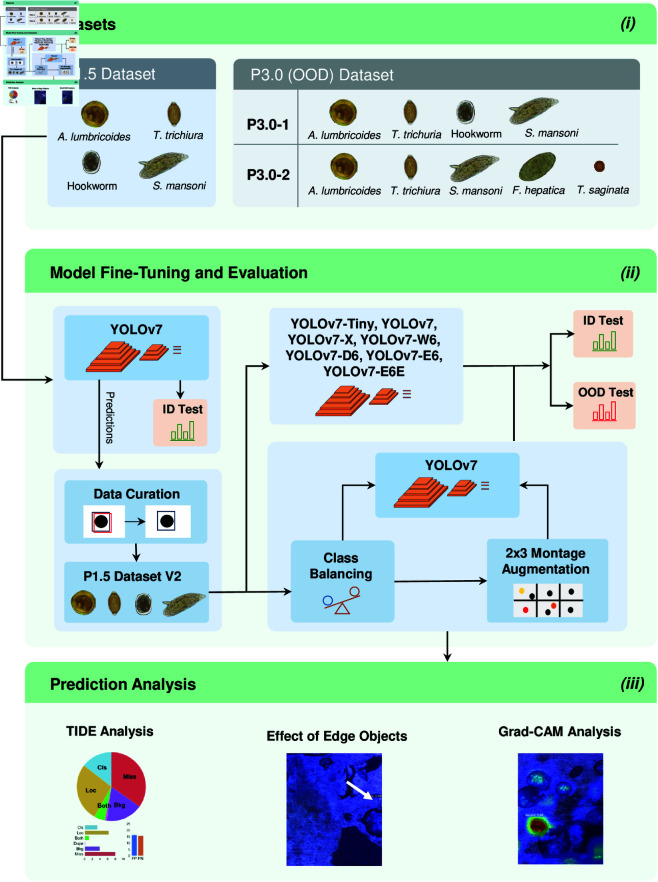
Overview of our research methodology through a general block diagram. (*i*) Datasets used in our research: the P1.5 dataset (collected by the P1.5 AI-DP prototype) is used for model fine-tuning, validation, and ID tests, and the P3.0 dataset (collected by the P3. AI-DP prototype) for OOD tests. (*ii*) Model fine-tuning and evaluation: The YOLOv7 model is first fine-tuned on the P1.5 dataset to create a curated dataset. All YOLOv7 variants are then fine-tuned using this curated dataset, followed by evaluation on ID and OOD test sets. In addition, class distribution balancing is applied to the curated dataset and subsequently a 2×3 montage augmentation is conducted to assess model robustness in OOD scenarios. (*iii*) Prediction analysis: Utilization of TIDE to identify six key error sources affecting mAP, analysis of edge object impact on performance, and adoption of Grad-CAM for visualizing activation maps of TP, FN, and FP predictions.

In this section, we provide a comprehensive breakdown of the primary elements of our research methodology. As depicted in Fig 1, our approach begins with a detailed description of our datasets, explaining their collection, significance and utilization in our study. Subsequently, we elaborate on our choice of SOTA object detection models, namely YOLOv7 and several of its variants. We then present a brief description of our experimental setup, alongside the metrics employed for evaluation. A key aspect of our methodology includes testing for OOD generalization, as well as employing detailed error analysis and interpretability techniques to identify and understand model failures. We also introduce a new augmentation scheme guided by prior knowledge to enhance model effectiveness under OOD conditions.

### 3.2. Dataset descriptions

The P1.5 dataset, which was previously made publicly available in [10], was derived from an experimental WSI scanner prototype (P1.5). It contains four types of parasite eggs collected from human KK stool smears: *Ascaris lumbricoides* (*A. lumbricoides*), *Trichuris trichiura* (*T. trichiura*), hookworm, and *S. mansoni*. The P3.0 dataset was collected using the latest version of the WSI prototype (P3.0) and includes six types of parasite eggs: *A. lumbricoides*, *T. trichiura*, hookworm, *S. mansoni*, *Fasciola hepatica* (*F. hepatica*), and *Taenia saginata* (*T. saginata*). While the P3.0 dataset is not yet publicly available, we were granted access to a specific subset for conducting generalizability testing of our AI models. It is important to note that the P1.5 dataset consists entirely of human samples, whereas the P3.0 dataset includes a small proportion of animal samples which were collected through controlled experiments at Ghent University. These samples were obtained from chimpanzees at Antwerp Zoo (Belgium), which were spiked with eggs from five distinct helminth species, including *A. lumbricoides*, *T. trichiura*, *S. mansoni*, *F. hepatica*, and *T. saginata*. The images were captured under varying experimental conditions, including different diaphragm apertures (open >20 mm, 8 mm, and 3 mm), scanning times (30 min, 50–60 min, and 24 h), and temperatures (25 ∘C and 35 C). Ethical considerations, including approval and informed consent for human samples, are detailed in the Ethics statement Sect 3.1 at the outset of this section. In contrast, the animal samples were collected from Antwerp Zoo and did not require ethical approval. This is because the samples were collected during the cleansing of the enclosures.

#### 3.2.1. Sample preparation.

For both the P1.5 and P3.0 datasets, stool samples were processed following the WHO guidelines for KK thick smears [6]. The process involved collecting a small, standardized amount of stool (41.7 mg) using a special template. The sample was then placed on a microscope slide and covered with a thin, absorbent strip pre-soaked in a special solution containing glycerol and a mild dye (malachite green). The glycerol helped clear the stool, making the parasite eggs easier to see, while the dye slightly stained the background to improve contrast. After waiting 30–60 minutes, the slide was examined under a microscope at 100× magnification to count the parasite eggs. The number of eggs was then used to calculate infection intensity, expressed as eggs per gram (EPG) of stool. A detailed explanation of the technique can be found in supporting information S1 Text for further reference. For AI-DP, each slide was labeled with a QR code containing key information, such as a unique slide number, sample details, study ID, and the date and time of preparation. This labeling allowed for automated sample recognition when inserted into a digital imaging system, making the process more efficient and reducing human error.

#### 3.2.2. Scanning protocol.

Slides that tested positive for helminth eggs were scanned to build a robust image database for AI training and performance assessment. Positive slides were initially identified using standard microscopy, and only those containing at least 20 eggs (approximately 480 EPG) were selected for scanning. This approach optimized the annotation process by ensuring that each scanned slide contributed a substantial number of eggs. Scanning was performed between 30 and 60 minutes after slide preparation to capture well-defined hookworm eggs, following established protocols [6]. However, due to field constraints and the prioritization of manual KK diagnostics for patient treatment, some slides were not scanned within this recommended time frame, with a few processed after 24 hours in accordance with guidelines for *S. mansoni* [6].

#### 3.2.3. Dataset preparation.

**Fig 2:**
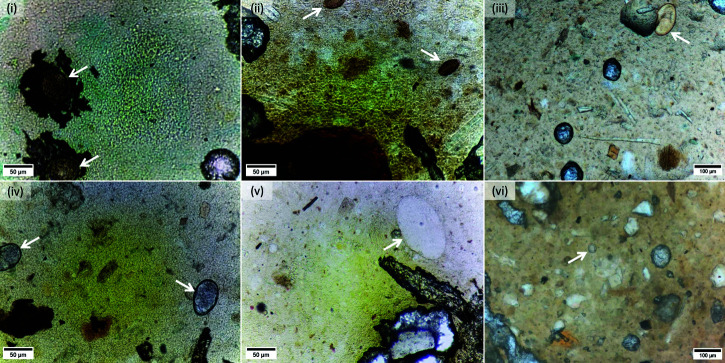
Sample images of eggs considered in our study. The first two columns depict the four types of helminth eggs in the P1.5 dataset, whereas the last column shows eggs from the P3.0 dataset (OOD test set): (*i*) two *Ascaris lumbricoides* eggs, (*ii*) two *Trchuris trichiura* eggs, (*iii*) a single *Fasciola hepatica* egg, (*iv*) two hookworm eggs, (*v*) a single *Sschistosoma mansoni* egg, and (*vi*) a single *Taenia saginata* egg.

**P1.5 dataset.** The P1.5 WSI scanner, using a camera system with a resolution of 1640 × 1232 pixels and a pixel size of ≈0.262 μm, was utilized to scan 300 freshly prepared KK stool smear samples, resulting in 7,780 distinct field-of-views (FOVs). Initially, domain experts annotated approximately 100 eggs from each of four classes: *A. lumbricoides*, *T. trichiura*, hookworm, and *S. mansoni*. Using these annotated FOVs, an object detection model was trained to automatically annotate additional FOVs. The predictions of the model were then rigorously verified by domain experts. This cycle of automated detection and expert verification was executed repeatedly, incorporating new FOVs for continual improvement. At the end, three types of STH eggs—*A. lumbricoides* (8,600), *T. trichiura* (4,083), and hookworm (3,623)—as well as 684 *S. mansoni* eggs were identified and annotated. Thus, the total count of all annotated eggs amounted to 16,990. Representative FOVs for each class type are depicted in the first and second columns of Fig 2, providing a visual overview of the four egg types included in our study.

The P1.5 dataset is utilized for fine-tuning, validating, and conducting ID testing of our models. In partitioning the P1.5 dataset, we adhered to the approach delineated in the benchmark paper [10]. The dataset was segregated into training, validation, and test sets. The specific distribution for each partition is presented in Table 1. This table shows the egg counts prior to and after data curation. Our experiments (those conducted following data curation) leveraged the curated data, the specifics of which are detailed in the results section.

**Table 1:** Dataset partition: Split of the P1.5 dataset into training, validation, and test sets, before and after data curation.

Class Names	Training		Validation		Test		All	
	Before	After	Before	After	Before	After	Before	After
*A. lumbricoides*	6,119	6,402	1,648	1,766	833	899	8,600	9,067
*T. trichiura*	2,839	2,928	859	904	385	406	4,083	4,238
Hookworm	2,514	2,601	721	760	388	412	3,623	3,773
*S. mansoni*	477	477	142	143	65	64	684	684
**All**	11,949	12,408	3,370	3,573	1,671	1,781	16,990	17,762

**P3.0 dataset.** The P3.0 WSI scanner, using a camera system with a resolution f 1280 × 720 and a pixel size of ≈0.93 μm, was utilized to collect FOVs containing six distinct types of helminth eggs: the four found in the ID dataset (*A. lumbricoides* , *T. trichiura*, hookworm, and *S. mansoni*), along with two additional types (*T. saginata* and *F. hepatica*). We further divided the P3.0 dataset into two subsets: P3.0-1 and P3.0-2. P3.0-1 includes only FOVs that contain egg types present in the ID dataset and is derived primarily from human samples with a small mixture of animal samples. This subset is designed to assess the effectiveness of the models when transitioning from the P1.5 to P3.0 acquisition device. The second subset, P3.0-2, also encompasses parasite egg types that were not included in the model fine-tuning phase. This subset is derived entirely from animal samples and devised to evaluate the indifference nature of the models when they encounter parasite egg categories that were not included during fine-tuning.

The distribution of eggs in the subsets is as follows: within P3.0-1, there are 5,423 *A. lumbricoides*, 404 *T. trichiura*, 54 hookworm, and 134 *S. mansoni* eggs; within P3.0-2, there are 47 *T. saginata* and 201 *F. hepatica* eggs. Because of co-infections, there are also 125 *A. lumbricoides*, 6 *T. trichiura*, and 13 *S. mansoni* eggs. The last column of Fig 2 shows sample eggs of *F. hepatica* and *T. saginata*.

### 3.3. Model descriptions

#### 3.3.1. YOLO.

The YOLO series represents a collection of object detectors based on DCNNs, known for their unified approach. Unlike two-stage detectors such as Faster R-CNN, which require separate components for proposing bounding box regions and predicting class probabilities, YOLO models are trained end-to-end, jointly optimizing both bounding box regression and object classification in a single network. This allows them to predict multiple bounding boxes and class probabilities concurrently. Owing to their continuous advancements and success, they have emerged as the preferred method for object detection tasks in medical applications [23]. While YOLOv10 represents the latest advancement in the series at the time of writing, our experiments concentrated on YOLOv7, as it is accompanied by a detailed scientific paper [24], providing a more robust foundation for academic analysis and comparison. We are aware that subsequent versions might introduce new architectural improvements and/or optimization techniques. But, as they continue to build upon the core concepts of YOLO, the main conclusions and insights derived from our experiments with YOLOv7 will remain applicable, providing a relevant basis for understanding the performance and trade-offs of later versions.

#### 3.3.2. YOLOv7.

**Fig 3:**
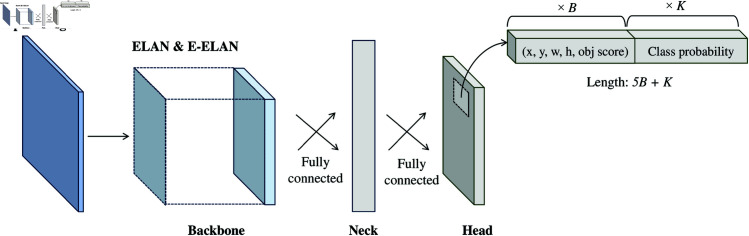
A typical high-level YOLOv7 architecture. The backbone extracts features from an image, the neck connects the backbone to the head and creates aggregated features maps by merging information from different scales and aspect ratios, and finally, the head takes the responsibility of predicting the bounding boxes and class probabilities [25].

Similar to other iterations of YOLO, YOLOv7 comprises three fundamental components, as shown in Fig 3, namely the backbone, the neck, and the head. The “backbone” is responsible for extracting representative features from the dataset; the “neck” generates feature pyramids to aggregate features from multiple scales to improve the models ability to handle objects with different sizes and aspect ratios, and connects the backbone to the “head”; and the “head” predicts bounding box values and class probabilities before passing them to the Non-Maximum Suppression (NMS) algorithm.

Internally, YOLO models create *S* × *S* grid cells to split the input image into tiles. The grid cell where the center of the ground truth bounding box falls is responsible for detecting the object. Each grid cell predicts *B* bounding boxes and their objectness score, along with their class predictions. Each bounding box is represented by four coordinates: *x* and *y*, which denote the center offsets of the object location, and *w* and *h*, which represent the width and height of the object, respectively. The objectness score provides an indication of the probability that an object is present within a particular grid cell. Once an object is detected, the next step is to predict the probability of *K* classes associated with the bounding boxes.

YOLOv7 not only matches the efficiency and effectiveness of current SOTA object detectors but also introduces substantial contributions. These include an advanced convolutional layer aggregation technique, referred to as the Extended Efficient Layer Aggregation Network (E-ELAN), and a novel model scaling method for concatenation-based models, known as compound model scaling. Furthermore, YOLOv7 is the first in the YOLO series to introduce the concept of a trainable bag-of-freebies (BoFs). Trainable BoFs refers to optimizing model architecture modules and training methods to improve model predictive performance, irrespective of the training cost, without increasing inference time. In YOLOv7, two major contributions are included as trainable BoFs: (1) planned parameterized convolution and (2) lead-guided and coarse-to-fine lead-guided label assigner. The first contribution, planned parameterized convolution, optimizes the convolutional layers by allowing them to adapt dynamically to different feature maps, improving feature extraction and representation. The second contribution, the lead-guided and coarse-to-fine lead-guided label assigner, refines the process of assigning labels to anchors by focusing on more informative and harder examples during training, thereby enhancing the model’s generalization capabilities.

#### 3.3.3. Variations of YOLOv7.

There are three main variations of YOLOv7 tailored to different GPU requirements: YOLOv7-Tiny for edge GPUs, YOLOv7 for standard GPUs, and YOLOv7-W6 for cloud GPUs. To produce YOLOv7-X, the newly proposed compound model scaling technique was incorporated into YOLOv7, along with stack scaling applied to the neck. In a similar fashion, the proposed compound model scaling was integrated into YOLOv7-W6 to derive its variants, namely YOLOv7-E6 and YOLOv7-D6. Subsequently, the newly introduced E-ELAN technique was applied to YOLOv7-E6, resulting in its most precise iteration, YOLOv7-E6E.

YOLOv7-Tiny, YOLOv7, and YOLOv7-X are P5 models, while the remaining are P6 models. ‘P’ levels represent the number of feature maps (pyramid levels). P5 models reserve P3 to P5 pyramid levels for detecting large, medium, and small-sized objects with downscaling strides of 32, 16, and 8, respectively, and take an input image resolution of 640*px* × 650*px*. Contrary, P6 models reserve P3 to P6 levels for detecting extra-large, large, medium, and small-sized objects with strides of 64, 32, 16, and 8, respectively, and take an input image resolution of 1280px×1280px.

### 3.4. Model fine-tuning

**Fig 4:**
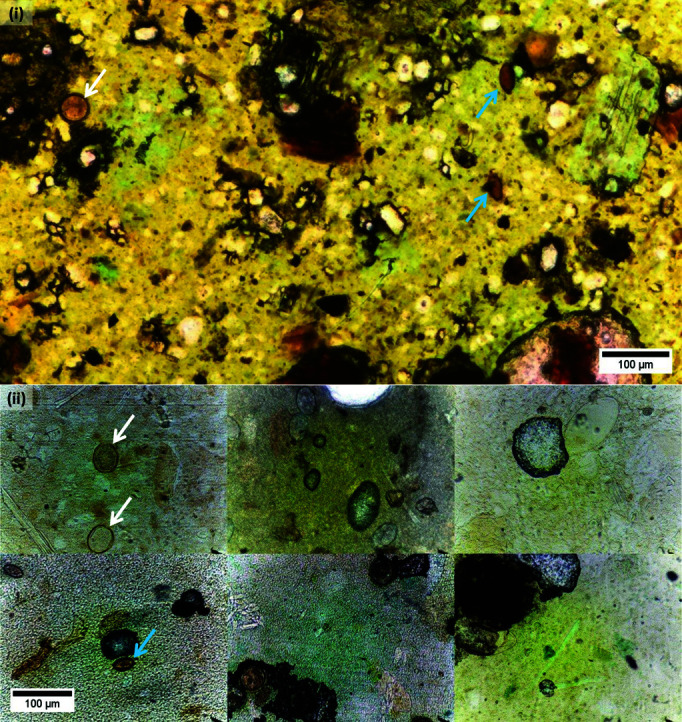
Montage data augmentation: A comparative visualization of (*i*) egg appearances in the out-of-distribution test dataset alongside (*ii*) the created 2×3 montage, highlighting the size of *Ascaris lumbricoides* (white arrow) and *Trichurs trichiura* (blue arrow) eggs.

**Data augmentation.** YOLOv7 employs mosaic data augmentation during batch generation as a default technique to increase the diversity of training samples and thereby increase the generalizability of the model. This method primarily focuses either on 2 × 2 or 3 × 3 configurations to satisfy the square image input requirement of the model. While mosaic augmentation followed by affine transformations (rotation, translation, scaling, etc.) enriches the training data, its reliance on square configurations limits its ability to fully address potential size variations encountered in OOD data that can also contain rectangular image shapes and/or eggs with smaller sizes compared to the ID test data. To tackle this challenge and be more robust for OOD scenarios, we propose a new pre-processing step involving 2 × 3 montage creation. Unlike the default mosaic augmentation technique, these montages are constructed prior to batch generation. Each montage randomly combines six FOVs into a single composite entity, with object sizes adjusted to resemble those found in the OOD dataset. During training, these montages also undergo the default data augmentation techniques, including the default mosaic augmentation. This combined approach effectively enriches the training data with variations in object size specifically to be more robust for OOD scenarios. Fig 4 provides a visual comparison of egg sizes in the OOD test set against our custom 2×3 montage.

**Fine-tuning settings.** In this study, we applied transfer learning and experimented with all variations of YOLOv7 to leverage the strengths of pre-existing models and accelerate the training process. We seeded the models with pre-trained weights trained on the MS COCO dataset [24], which provides a robust starting point due to its diverse object classes and extensive training data. Subsequently, we fine-tuned the models using the default hyperparameter settings, making adjustments only to the number of classes, epochs, and training batch size to tailor the models to our specific task. This approach aimed to maximize performance while minimizing the need for extensive training from scratch. To do so, the original classes hyperparameter in the YOLOV7 variants was adjusted from 80 to 4 to align with the number of parasite egg categories in our use case. While fine-tuning the YOLOv7-E6, -D6 and -E6E models, we additionally decreased the batch size from the default 16 to 8 because of compute resource limitations. Furthermore, to mitigate the risk of overfitting, given the limited number of training samples, the default number of epochs was adjusted from 300 to 100. The full hyperparameter settings are provided in the supporting information S1 Data. The learning curves corresponding to these adjustments are available in the supporting information S1 Fig.

**Computational environment.** All experiments were carried out in the JupyterHub cloud [26] (accelerated by an NVIDIA A40 GPU) of Ghent University-imec.

### 3.5. Performance evaluation metrics

To assess the correctness of model predictions, we utilized four widely recognized evaluation metrics: recall, precision, mean average precision at an intersection over union (IoU) threshold of 0.5 (*mAP*@*IoU*_*0.5*_), and the F1-score. These metrics are based on true positive (TP), false negative (FN), and false positive (FP) predictions. Precision quantifies the proportion of correctly identified items as a ratio of TP to the sum of TP and FP (Precision=TP/(TP  +  FP)), reflecting the ability of the model to minimize FP predictions. Recall measures the proportion of correctly identified relevant items as a ratio of TP to the sum of TP and FN (Recall=TP/(TP  +  FN)), indicating the capacity of the model to minimize FN predictions. The F1-score, or the harmonic mean of precision and recall, provides a balanced view of both metrics, without overly emphasizing either FN or FP predictions. This score is calculated as 2*(Precision*Recall)/(Precision+Recall). Finally, *mAP*@*IoU*_0.5_ evaluates the average precision across classes at an IoU threshold of 0.5, representing the area under the precision-recall curve for correctly detected objects. It is expressed as $\frac{1}{n}\sum_{i=1}^{n}{\text{AP@IoU}}_{0.5}^{\left(i\right)}$, where *n* is the number of classes.

### 3.6. Prediction analysis

Our approach also includes the use of SOTA tools for investigating error dynamics and visually explaining the decision-making process of the models. To achieve this, we use TIDE and Grad-CAM. The former helps us to categorically dissect the errors, providing insights into specific areas where the model may be underperforming, such as localization or classification errors—this allows to target a specific error type. The latter visualizes the heatmaps of the detected eggs based on the gradient weights from the detection layers, offering a visual representation of which parts of the image are contributing most to the predictions of the models—this helps in understanding the focus of the model and ensuring that it is making decisions based on relevant features.

**TIDE:** TIDE is a general tool to quantify the drop in mAP due to six types of object detection and instance segmentation errors: classification errors (cls), localization errors (loc), both classification and localization errors (both), duplicate detections (dupe), background errors (bkg), and missed ground truth errors [27]. TIDE can be applied directly to the predictions of object detection models without requiring any modifications to the underlying model. This ease of integration makes it convenient to use within existing workflows. Furthermore, TIDE provides meaningful quantification of errors and fully isolates error categories, which is essential for planning effective rectification procedures.

**Grad-CAM:** Grad-CAM constitutes a class-discriminative localization technique specifically tailored for CNN-based computer vision models. This technique serves the purpose of visualizing and comprehending the decision-making process employed by convolutional neural networks in computer vision tasks. Through the generation of informative heatmaps, Grad-CAM highlights the regions within an input image that hold critical significance for model predictions, effectively revealing the areas where a model directs its focus when arriving at a decision.

Fundamentally, Grad-CAM leverages the gradients of the target class in relation to the final convolutional layer, which resides prior to the decision layer, in the CNN architecture. These gradients serve as indicators of the importance assigned to each feature map during the final object determination. A higher gradient value indicates a more substantial contribution of the corresponding feature map to the prediction process. Grad-CAM facilitates an insightful understanding of the decision-making rationale of a neural network, giving insight into the areas of interest within the input image that play a pivotal role in model decisions [28].

Grad-CAM is commonly implemented for the generic analysis of classification tasks. However, object detection models output dictionaries containing bounding boxes, labels, and scores, making it challenging to compute the gradients in a generic manner. Customizing the models for Grad-CAM analysis often becomes necessary. For our experiments, we leveraged an implementation of Grad-CAM that is available through a GitHub repository [29], which allowed us to effectively adapt and compute the gradients for visualization purposes.

## 4. Results

In this section, we present the outcomes obtained from seven experiments.

**Experiment 1: Data curation.** We initially experimented with the basic YOLOv7 model, maintaining the default hyperparameter configurations, except for adjusting (1) the original classes hyperparameter from 80 to 4 to encompass only the four classes relevant to our use case and (2) the number of epochs from 300 to 100 to fit our available computational resources and avoid overfitting. Changing from the R-FCN model of the original paper to YOLOv7 here gave no significant change in effectiveness [10]. Table 2 summarizes the effectiveness achieved by the basic YOLOv7 model in this initial experiment.

**Table 2:** Effectiveness of the basic YOLOv7 model before data curation. The basic YOLOv7 model was fine-tuned and evaluated using the P1.5 dataset (before it underwent thorough data curation). This model was employed to subjectively inspect FP and FN predictions originating from the training, validation, and test sets. Both precision and recall values were calculated using an IoU threshold of 0.5, along with the confidence score corresponding to the highest F1-score.

Class Names	Counts		Precision (%)		Recall (%)		F1-score (%)		AP@IoU_0.5_ (%)	
	Val	Test	Val	Test	Val	Test	Val	Test	Val	Test
*A. lumbricoides*	1648	833	95.58	96.18	97.69	97.36	96.62	96.77	98.69	98.68
*T. trichiura*	859	385	95.50	95.65	96.97	97.12	96.23	96.38	97.72	98.23
Hookworm	721	388	95.90	96.93	97.43	97.64	96.66	97.28	98.64	98.98
*S. mansoni*	142	65	97.18	96.84	97.18	94.45	97.18	95.63	98.22	95.52
**Weighted Average**	3370	1671	95.70	96.26	97.43	97.26	96.55	96.75	98.41	98.52

**Fig 5:**
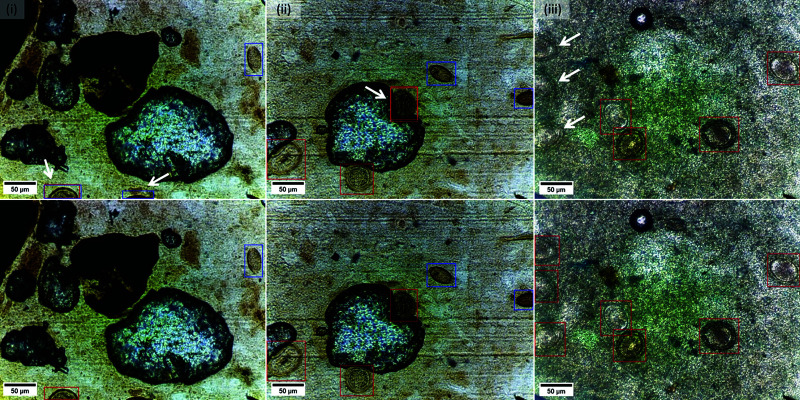
Errors detected in the ground truth. The first row shows the ground truth annotations, while the second row shows the basic YOLOv7 model predictions. The red bounding boxes delineate *A. lumbricoides* eggs and the blue bounding boxes delineate *T. trichiura* eggs. A white arrow denotes the location of an identified error: (*i*) an *A. lumbricoides* egg that has been assigned to both the *A. lumbricoides* and *T. trichiura* classes (left) and a barely identifiable edge object that has been annotated as a *T. trichiura* egg (right), (*ii*) an *A. lumbricoides* egg that comes with duplicated annotations, and (*iii*) three *A. lumbricoides* eggs that were missed during annotation.

Suspecting potential annotation errors in the ground truth, we pull all the FOVs in the training, validation, and test sets that contributed to the FN and FP predictions. In this way, we were able to identify several issues, as shown in Fig 5. Assisted by a domain expert, i.e. a parasitologist, we subsequently evaluated and curated all the issues related to FN and FP predictions manually by doing a side-by-side comparison of the prediction-ground truth pairs. Some of the FN prediction errors originated from duplicated annotations, either of the same class or different classes, and barely recognizable cropped but annotated edge objects. In contrast, several FP prediction errors originated from missing eggs during annotation and from mistakenly annotating edge objects that resemble the eggs of the target parasite. Note that the edge objects are difficult to annotate because they are cropped at the edges of the FOVs and do not provide complete information to make educated decisions.

To address the issue of duplicated annotations, we removed the extra bounding boxes if the class labels were the same. If the extra bounding box belonged to a different class, a parasitologist was consulted to decide which of the duplicate bounding boxes was correct and to remove the other one. To address the remaining errors, the parasitologist went through each instance and provided the following comments: “an egg”, “not an egg”, “probably an egg”, “probably not an egg”, and “I cannot make a call”. When introducing the second version of the dataset (P1.5 V2), we added all instances identified as “an egg” and “probably an egg” to their respective classes and rejected the rest. We present the distribution of the eggs after data curation in Table 1. The curated dataset (P1.5 V2), along with the P3.0 OOD subsets, is available at https://doi.org/10.34740/kaggle/ds/5845499.

Thanks to the curation step, a small improvement in the weighted average of F1-score was achieved by 0.88% and 0.39% for the validation and test datasets, respectively, as can be calculated from Tables 2 and 3.

**Table 3:** Prediction effectiveness of YOLOv7 and several variants on the curated dataset. Comparative prediction effectiveness of YOLOv7 and several variants on the validation and test sets of the curated P1.5 dataset (P1.5 V2). Both precision and recall values were calculated using an IoU threshold of 0.5, along with the confidence score corresponding to the highest F1-score. For ease of comparison, evaluation metric values from the benchmark paper are added at the top.

Class Names	Counts		Precision (%)		Recall (%)		F1-score (%)		AP@IoU_0.5_ (%)	
	Val	Test	Val	Test	Val	Test	Val	Test	Val	Test
**Benchmark (R-FCN) [10]**										
*A. lumbricoides*	-	833	-	95.40	-	95.90	-	95.60	-	-
*T. trichiura*	-	385	-	94.20	-	96.40	-	95.30	-	-
Hookworm	-	388	-	95.00	-	97.70	-	96.30	-	-
*S. mansoni*	-	65	-	91.80	-	86.20	-	88.90	-	-
**Weighted Average**	-	1,671	-	94.90	-	96.10	-	95.40	-	-
**YOLOv7-Tiny**										
*A. lumbricoides*	1,766	899	97.10	96.92	97.85	98.12	97.47	97.52	99.03	99.51
*T. trichiura*	904	406	95.91	95.76	97.46	98.28	96.68	97.00	98.45	99.09
*Hookworm*	760	412	97.66	95.69	99.08	97.09	98.37	96.39	99.55	98.19
*S. mansoni*	143	64	97.17	96.92	96.21	98.44	96.69	97.67	98.29	98.75
**Weighted Average**	3,573	1,781	96.92	96.36	97.95	97.94	97.43	97.14	98.96	99.08
**YOLOv7**										
*A. lumbricoides*	1,766	899	97.92	98.06	96.32	97.55	97.11	97.80	98.67	98.70
*T. trichiura*	904	406	98.11	96.25	96.57	97.29	97.33	96.77	98.49	97.85
*Hookworm*	760	412	98.71	97.09	97.63	97.09	98.17	97.09	99.24	98.39
*S. mansoni*	143	64	97.24	97.45	98.60	98.44	97.91	97.94	98.94	98.89
**Weighted Average**	3,573	1,781	98.11	97.40	96.75	97.42	97.42	97.41	98.76	98.44
**YOLOv7-X**										
*A. lumbricoides*	1,766	899	95.05	96.93	96.73	94.44	95.88	95.67	98.24	98.90
*T. trichiura*	904	406	93.20	97.47	94.06	91.13	93.63	94.19	96.79	96.06
Hookworm	760	412	97.07	96.18	95.79	91.70	96.43	93.89	97.23	95.67
*S. mansoni*	143	64	88.35	94.16	90.20	79.69	89.27	86.32	90.66	90.70
**Weighted Average**	3,573	1,781	94.74	96.78	95.59	92.52	95.16	94.58	97.35	97.21
**YOLOv7-W6**										
*A. lumbricoides*	1,766	899	96.79	96.23	96.04	96.54	96.41	96.39	98.36	98.64
*T. trichiura*	904	406	94.88	91.49	94.80	95.29	94.84	93.35	97.23	96.76
Hookworm	760	412	97.16	95.35	95.79	94.54	96.47	94.94	98.00	96.60
*S. mansoni*	143	64	92.60	90.85	91.61	93.75	92.10	92.28	95.36	93.89
**Weighted Average**	3,573	1,781	96.22	94.75	95.56	95.69	95.85	95.21	97.88	97.57
**YOLOv7-E6**										
*A. lumbricoides*	1766	899	96.59	95.92	96.35	96.77	96.47	96.34	98.29	98.13
*T. trichiura*	904	406	96.09	93.05	94.58	95.69	95.33	94.35	97.05	97.37
Hookworm	760	412	96.84	94.43	96.89	95.63	96.86	95.02	98.12	96.56
*S. mansoni*	143	64	92.99	93.55	93.01	90.62	93.00	92.06	95.10	91.65
**Weighted Average**	3,573	1,781	96.37	94.84	95.88	96.04	96.13	95.43	97.81	97.36
**YOLOv7-D6**										
*A. lumbricoides*	1766	899	97.64	98.30	96.07	96.70	96.85	97.49	98.40	98.57
*T. trichiura*	904	406	97.29	97.98	95.35	95.56	96.31	96.75	97.44	97.33
Hookworm	760	412	98.13	96.46	96.58	94.42	97.35	95.43	98.46	96.63
*S. mansoni*	143	64	96.50	97.86	96.48	95.31	96.49	96.57	96.89	96.36
**Weighted Average**	3,573	1,781	97.61	97.79	96.01	95.86	96.81	96.81	98.11	97.76
**YOLOv7-E6E**										
*A. lumbricoides*	1766	899	97.51	97.98	97.62	96.99	97.57	97.48	98.68	98.36
*T. trichiura*	904	406	96.90	97.54	96.81	97.5	96.86	97.52	98.33	98.38
Hookworm	760	412	98.08	98.27	97.63	96.56	97.86	97.41	98.79	98.34
*S. mansoni*	143	64	92.10	96.64	97.87	98.44	94.90	97.53	97.66	98.64
**Weighted Average**	3,573	1,781	97.26	97.90	97.43	97.06	97.35	97.47	98.57	98.37

**Experiment 2: Effectiveness of YOLOv7 variants on the curated dataset.** Table 3 presents a comprehensive evaluation of the predictive effectiveness of YOLOv7 and different variants after data curation. We can observe that the standard YOLOv7 model designed for regular GPUs and the YOLOv7-E6E model designed for cloud GPUs emerge as notably stable across all metrics. On the ID test set, these models obtain a weighted average precision of 97.40±0.66% and 97.90±0.35%, a recall of 97.42±0.27% and 97.06±0.42%, an F1-score of 97.41±0.45% and 97.47±0.04%, and an mAP@IoU_0.5_ of 98.44±0.35% and 98.37±0.05%, respectively. Contrary, the YOLOv7-X model marked lower prediction effectiveness, with a weighted average precision of 96.78±0.67%, a recall of 92.52±2.89%, an F1-score of 94.58±1.79%, and an mAP@IoU_0.5_ of 97.21±1.95%. In terms of individual metric performance on the test set, the YOLOv7-E6E achieved the highest weighted average precision and F1-score, whereas YOLOv7-Tiny showcased the best weighted average recall of 97.94±0.47% and an mAP@IoU_0.5_ of 99.08±0.53%. Despite these variations in prediction effectiveness, each model variant showed notably high effectiveness when evaluated using the curated P1.5 dataset (named P1.5 v2): the scores obtained were comparable to those in the original article [10], with only a small improvement in the weighted average of the F1 score as a result of curation (see Experiment 1), having precision, recall, and F1-score values of 94.90±0.80%, 96.10±2.1%, and 95.40±1.4%, respectively. The prediction effectiveness of YOLOv7-Tiny is particularly noteworthy when taking into account model weight size and architectural complexity (approximately 27× less than the most precise and architecturally complex variation, YOLOv7-E6E), especially given its adaptability for edge devices.

**Experiment 3: Robustness in OOD generalization.** In the domain of computer vision, a key challenge is ensuring the generalization of models to OOD data, where models are evaluated on data drawn from a different distribution than the one encountered during training. This scenario is important for assessing the robustness of models in real-world deployment, where input data characteristics can shift due to environmental changes, acquisition device variations, or other factors, while the underlying task and desired outputs remain the same [30]. This experiment investigates this challenge in detail using two distinct datasets: P3.0-1 and P3.0-2. These datasets present inherent variations in resolution and magnification attributable to changes in acquisition devices compared to the P1.5 dataset, particularly in the camera subsystem. The P1.5 WSI prototype used the Raspberry Pi Camera Module 2, whereas the P3.0 WSI prototype used the e-CAM24_CUNX camera.

**Fig 6:**
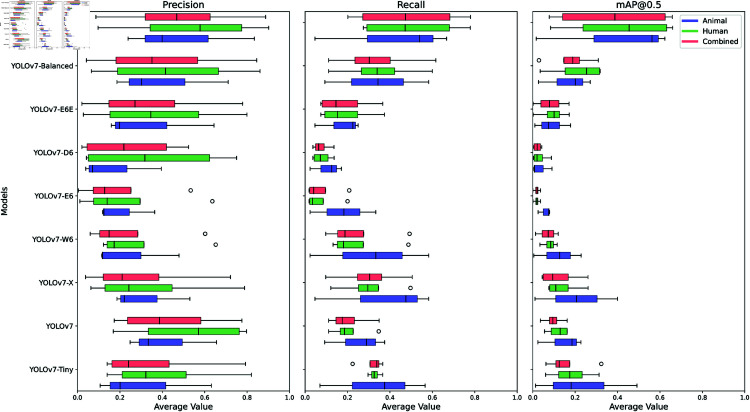
Robustness in OOD generalization: change in capturing device. Box-and-whisker plots illustrate the performance of YOLOv7 and its variants across the precision, recall, and mAP metrics, giving insight into OOD generalization as the capturing device transitions from P1.5 to P3.0 WSI. The plots also illustrate the effectiveness of the models for the human and animal samples separately, as well as their combined impact.

The P3.0-1 dataset is arranged to evaluate OOD generalization in scenarios where there is a change in capturing devices, while the underlying parasite eggs remain similar to those in the ID dataset. We assessed the effectiveness of YOLOv7 and several of its variants on this OOD dataset, as depicted visually through box-and-whisker plots in Fig 6. Our findings indicate that, with the exception of YOLOv7-Montage, all model variants struggle to generalize effectively.

Given the distinct data sources (animal and human samples), one might question whether the decline in model performance stems from sample type differences. However, as illustrated in Fig 6, while human samples exhibit a slightly higher average effectiveness across most models, the difference is not statistically significant compared to animal samples. This suggests that factors beyond sample origin, primarily the change in device, contribute to the observed generalization challenges.

**Fig 7:**
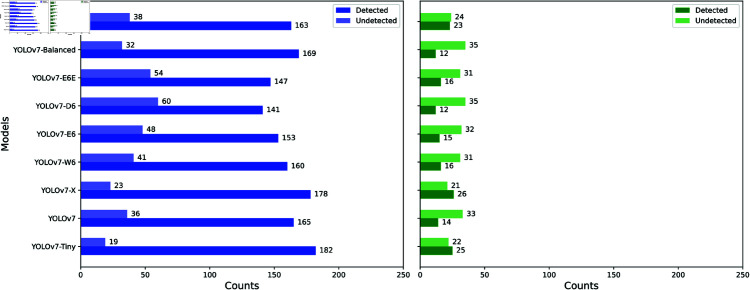
Robustness in OOD generalization: being indifferent to unknown egg types. Bar charts depicting the effectiveness of YOLOv7 and several variants in being indifferent to *F. hepatica* (left) and *T. saginata* (right) eggs. Detected refers to instances where the model misclassified the two egg types as one of the four ID classes. Undetected refers to instances where the model did not identify or classify the two egg types, which was the desired behavior.

Our OOD generalization assessment also involves testing models on datasets that contain classes not present in the ID dataset. This aspect of testing is particularly important for our study, given the diversity of helminth eggs that can be encountered in real-world scenarios, which closely resemble helminth eggs in the ID training set but were not explicitly included in it [31]. Specifically, we used the P3.0-2 OOD dataset, which contains two previously unseen parasite egg classes: *F. hepatica* and *T. saginata*. The bar graphs of Fig 7 show the effectiveness of the different YOLOv7 models in maintaining indifference to *F. hepatica* and *T. saginata* eggs. These bar graphs highlight a pronounced challenge faced by all tested models. Initially we assumed that, when the models are presented with FOVs containing only *F. hepatica* and *T. saginata*, and no other egg types, they would effectively consider the FOVs as egg-negative, showing no detections. However, contrary to our assumption, the models misidentify *F. hepatica* and *T. saginata* as one of the ID classes. This is particularly evident with *F. hepatica* eggs, where the ratio of incorrectly detected eggs to those undetected is significantly high (ranging from $\frac{141}{60}$ for YOLOv7-D6 to $\frac{182}{19}$ for YOLOv7-Tiny), indicating a substantial deviation from our expected effectiveness assumptions. Although the detection of *T. saginata* eggs shows a relatively more favorable proportion compared to *F. hepatica*, it is worth noting that the absolute counts of incorrectly detected *T. saginata* eggs as ID eggs are significant (the lowest being 12 for YOLOv7-D6 and YOLOv7-Balanced and the highest being 26 for YOLOv7-X, out of a total of 57 eggs).

**Fig 8:**
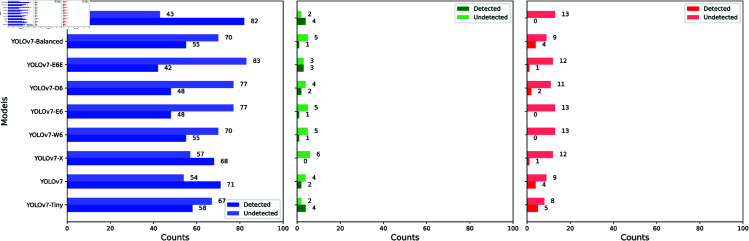
Number of ID parasite eggs in the OOD dataset. Bar charts depicting the effectiveness of YOLOv7 and several variants in detecting parasite eggs from the ID dataset when present as co-infections in the OOD dataset. *A. lumbricoides* (left), *T. trichiura* (middle), and *S. mansoni* (right). Detected refers to instances where the model correctly identified the four ID egg types present in the OOD dataset, as intended. Undetected refers to instances where the model failed to identify the four ID egg types in the OOD dataset.

Due to the presence of co-infections, the FOVs in the P3.0-2 OOD dataset also contain three types of parasite eggs that are found in the ID (P1.5 V2) dataset: *A. lumbricoides*, *T. trichiura*, and *S. mansoni*. Fig 8 shows the TP and TN prediction counts obtained by YOLOv7 and its several variants for these three parasite egg types. Analogous to the effectiveness observed on the P3.0-1 OOD test dataset, the investigated models demonstrate suboptimal detection of the three ID egg types, except for YOLOv7-Montage.

**Experiment 4: 2 × 323 montage augmentation.** In response to the low robustness against OOD data generated by the adoption of a new slide scanner and previously unseen co-infections, we employed and evaluated the effectiveness of the 2 × 3 montage data augmentation technique previously explained in Sect 3.4. We modified the training set from the P1.5 V2 dataset to achieve class balance by evenly distributing parasite eggs. All FOVs containing *S. mansoni* parasite eggs were included. FOVs from the remaining classes were selected randomly, using the number of *S. mansoni* FOVs as an upper limit. One important observation was the egg count variability across FOVs. This variation can be attributed to differences in egg densities. Notably, *A. lumbricoides* eggs often outnumber other types within a single FOV, followed by Hookworm and *T. trichiura*. For example, the average number of eggs per image in the training set of the P1.5 V2 dataset is as follows: *A. lumbricoides* (2.32), *T. trichiura* (1.57), Hookworm (1.72), and *S. mansoni* (1.02). The prevalence of co-infections also influences these counts. A comprehensive distribution of eggs after data balancing can be found in Table 4. Following this, we applied the proposed augmentation method to the balanced dataset.

**Table 4:** Dataset balancing. The distribution of FOVs and egg counts before and after balancing.

Classes	FOVs		Egg Counts	
	Before	After	Before	After
*A. lumbricoides*	2,757	801	6,402	1,794
*T. trichiura*	1,864	518	2,928	843
Hookworm	1,511	467	2,601	816
*S. mansoni*	466	466	477	477

After implementing our data augmentation strategy, we conducted two distinct experiments using the basic YOLOv7 model: the first experiment utilized the balanced dataset alongside default augmentation techniques, while the second experiment incorporated images generated by the proposed 2 × 3 montage augmentation technique. As shown in Table 5, both YOLOv7 models maintained effectiveness comparable to that of YOLOv7 on the unbalanced, full dataset, particularly when assessed against the designated ID validation and test sets. Specifically, the proposed augmentation technique enhanced the effectiveness for the OOD generalization scenario that involves a change in capturing device. As depicted in Fig 6, YOLOv7-Montage stands out with a notably higher median precision and significantly elevated median scores in recall and *mAP*@*IoU*_0.5_. This enhanced effectiveness is further highlighted by comparative data, such as those presented in Table 5, which demonstrates that compared to a model fine-tuned using the balanced dataset alone, the newly introduced augmentation technique yields increases of 8% in precision, 14.85% in recall, and 21.36% in *mAP*@*IoU*_0.5_. However, a crucial observation underscored by the bar charts in Fig 7 points to a substantial issue: the proposed augmentation strategy cannot adequately address the second OOD scenario in which the test set contains unseen parasite eggs.

**Table 5:** Effectiveness of 2 × 3 montage augmentation. Effectiveness comparison of YOLOv7 before and after montage augmentation across the ID validation and test sets, and an OOD test set, with a focus on the OOD scenario that involves a change in devices.

Augmentation	Precision (%)			Recall (%)			mAP@IoU_0.5_ (%)		
	Val P1.5	Test P1.5	Test P3.0-1	Val P1.5	Test P1.5	Test P3.0-1	Val P1.5	Test P1.5	Test P3.0-1
Default	98.55	97.33	39.72	96.66	97.65	33.33	97.58	97.67	16.14
Default + Montage	96.87	97.71	47.74	96.38	95.34	48.18	96.79	96.12	37.50

**Fig 9:**
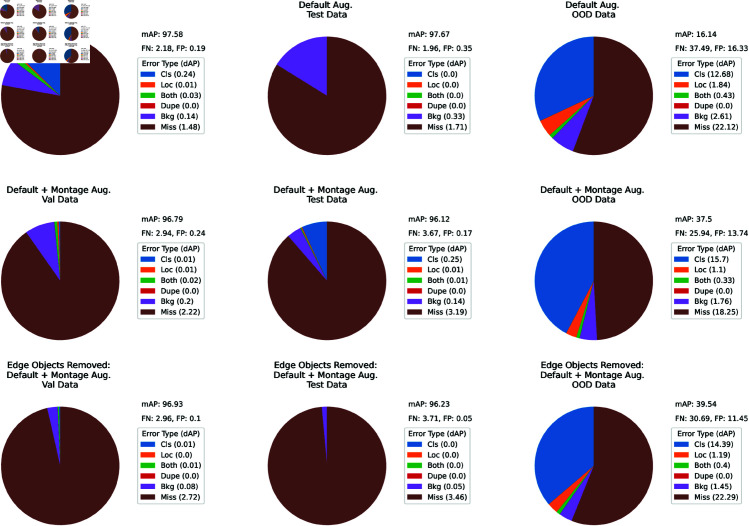
TIDE analysis. The pie charts illustrate the decline in mAP due to six distinct types of errors (cls, loc, both, dupe, bkg, and miss) across different validation and test sets. The prediction effectiveness of basic YOLOv7 models is compared when assessed with ID (P1.5 V2) test and validation datasets, as well as a OOD (P3.0-2) test dataset. The top row represents models fine-tuned on a balanced dataset. The middle row shows models fine-tuned incorporating the 2×3 montage image augmentation technique before the removal of edge objects, while the bottom row depicts the effectiveness of models after removal of the edge objects.

**Experiment 5: TIDE analysis.** For our analysis of the causes of reduced mAP values, the TIDE toolkit was instrumental in dissecting common errors into six categories, as depicted in Fig 9. This figure presents comparative visualizations, highlighting the error types derived from the ID validation and test sets, as well as the P3.0-1 OOD dataset. Specifically, the first row of pie-charts represents errors generated by the basic YOLOv7 model fine-tuned using the balanced dataset, while the subsequent row of pie-charts showcases outcomes following the integration of our proposed 2×3 montage augmentation technique.

**Fig 10:**
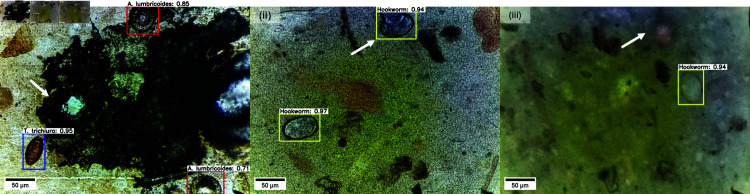
Missed eggs: The white arrows indicate the eggs missed by the model during detection. (i) Severely occluded *T. trichiura* egg by an artifact, (ii) two overlapping hookworm eggs detected as one, and (iii) an out-of-focus hookworm egg during FOV acquisition.

The incorporation of montage augmentation into the balanced dataset led to a slight decrease in the mAP of the basic YOLOv7 model when evaluated using the ID validation and test sets. Interestingly, this marginal decrease contrasts considerably with the pronounced improvement obtained for the P3.0-1 OOD test set. A consistent trend that can be identified in the ID validation and test sets was the prevalence of ‘missed eggs’ as the principal contributor to the aggregated errors. A more in-depth analysis revealed that these instances could be attributed to rare scenarios such as severe occlusions, detection of overlapping eggs as a single egg, and out-of-focus eggs during FOV acquisition, as illustrated in the sample images in Fig 10.

In relation to the ID validation and test sets, the occurrence of other error variants was found to be nominal, likely due to the rigorous curation processes we conducted earlier. However, in our assessment of OOD generalization using the P3.0-1 dataset, these errors were significantly more pronounced. This increase can be directly attributed to the absence of similar curation measures and the distinct differences introduced by the change in capturing device. In this context, ‘cls’ errors emerged as the second most significant factor contributing to reduced mAP values, surpassed only by the previously mentioned ‘missed eggs’.

**Experiment 6: Handling edge objects.** Domain experts rely on clear visualizations of objects within the FOV to determine whether an object belongs to a specific egg class. When they encounter severely cropped eggs at FOV edges, they may choose to abstain from making a decision due to limited visual information. In contrast, modern object detectors, equipped with extensive feature learning capabilities, can readily identify and classify these cropped eggs. However, this difference in approach can lead to a decrease in model effectiveness, as the model lacks the intuitive discretion of experts to abstain from uncertain classifications. Consequently, the model may introduce more false positives or misclassifications when handling edge-cropped eggs, impacting its reliability in practical applications. To address this issue, we implemented a post-processing strategy to drop all FP instances that occur due to edge objects when quantifying model effectiveness. This strategy included the adoption of an absolute criterion for determining whether an ‘edge egg’ should be dropped or not: the ‘edge egg’ must be aligned to the edge of the FOV with a maximum offset of 10 pixels. To investigate the impact of the proposed strategy, we employed the basic YOLOv7 model fine-tuned using the dataset that incorporated the proposed montage augmentation technique. As illustrated in Fig 9, the TIDE analysis offers insightful revelations: notably, the proportion of background (Bkg) errors observed in the pie-charts experiences a reduction upon the application of the suggested edge object removal strategy. Given the highly curated nature of the dataset, the quantitative effect of this strategy on the ID validation and test sets appears marginally modest, with mAP exhibiting an improvement of 0.14% and 0.11% for the ID validation and test sets, respectively. However, a considerable improvement was observed in the context of the P3.0-1 dataset, where the application of the edge object removal strategy coincides with a substantial 2% increase in mAP.

**Fig 11:**
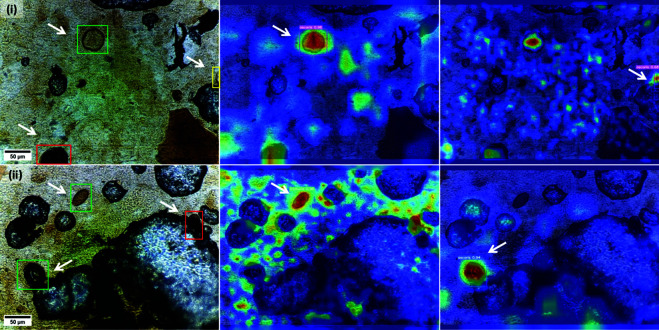
Grad-CAM heatmaps obtained for sample images. The first column depicts TP (green bounding box), FP (yellow bounding box), and FN (red bounding box) detections in two sample images (i) and (ii). The next two columns visualize areas where activation occurs when individual eggs (indicated by arrows) are detected.

**Experiment 7: Grad-CAM analysis.** Based on our subjective observations, we conducted a detailed visual examination, focusing especially on FP predictions prompted by edge objects and FN predictions caused by artifacts occluding eggs. To comprehend the gradient activation processes of the basic YOLOv7 model during decision-making, we employed Grad-CAM on two FOVs from the ID test set, as illustrated in Fig 11.

Sample image (*i*) in Fig 11 features *A. lumbricoides* eggs exclusively. The corresponding activation map indicates that, upon detecting the TP-annotated *A. lumbricoides* egg, there is minor activation in the area of the FN detection, even though the egg is substantially obscured by an external artifact. This suggests that while the model acknowledges certain *A. lumbricoides* features, it fails in decision-making due to incomplete information. Surprisingly, the absence of activation around the FP detection defies expectations, challenging the assumption that activation should be present near the edge object upon TP egg detection. Indeed, when the FP object (at the edge) is identified, the original assumptions hold: there is strong activation for the TP egg and a fainter response for the FN egg.

Sample image (*ii*) in Fig 11 comprises two TP detections (*A. lumbricoides* and *T. trichiura* eggs) and a FN detection (*T. trichiura egg*). The FN-annotated *T. trichiura* egg, significantly covered by an artifact, justifies why it is not detected by our model. Nonetheless, the accompanying activation map unveils intense activation on the marginally visible egg fraction. The disparity in activation between the obscured and exposed regions attests to the ability of the model in isolating fundamental egg attributes. This phenomenon is amplified in the TP-annotated *A. lumbricoides* egg case, where the non-obscured egg region clearly communicates the eggs class. The model identifies this with ease, and the activation expansively encompasses the anticipated egg territory.

## 5. Discussion

Our comprehensive study of YOLOv7 and its variants for detecting selected parasite eggs in microscopy images of stool smears presents an in-depth OOD analysis in this domain. To the best of our knowledge, we are the first to do such an investigation and it has uncovered several critical insights that impact the practical adoption of AI or machine learning solutions, particularly highlighting challenges in generalization and robustness when models encounter unseen conditions and classes. These insights, along with some of their practical implications for biomedical imaging, are discussed below in more detail.

**Model complexity.** Our study demonstrates that the effectiveness of the advanced YOLOv7-E6E model is on par with the effectiveness of its less complex counterparts, YOLOv7 and YOLOv7-Tiny, challenging the assumption that greater complexity leads to superior effectiveness. This observation suggests that for tasks involving a limited number of classes, model complexity may not be the primary determinant of effectiveness. Instead, the specificity—the degree to which training data faithfully mirror real-world scenarios encountered during model deployment—and the appropriateness—including but not limited to labeling and annotation accuracy—of the training set surface as important factors. However, further validation across diverse use cases is necessary to confirm this statement. The pre-trained YOLOv7 model and several of its variants were trained using a dataset encompassing nearly 80 classes, contrasting with our dataset of merely four classes. This disparity implies that the more sophisticated models might be over-parameterized for our specific task, negating the benefits usually gained from increased complexity. This insight is key to the development of optimized, task-specific models that are streamlined for practical deployment, particularly in edge computing scenarios.

**Robustness in uncontrolled environments.** The brittleness of models in the face of OOD data highlights a critical shortfall in current methodologies. Although our models excel on the ID validation and test sets, their effectiveness deteriorates when confronted with data variance, such as a change in capturing device or the introduction of new egg types. This points to a gap between success in a controlled environment, like a lab, and real-world applicability. This discrepancy underscores the need to move beyond conventional validation techniques by integrating tests that simulate real-world unpredictability. In particular, developers must consider environmental and contextual diversity during model creation, such as adequately incorporating ‘not an egg’ and ‘unknown egg’ categories, and considering KK sample preparation quality to ensure reliability and robustness upon deployment. This approach, however, requires multidisciplinary consensus among computer vision experts, hardware designers, platform developers, and domain experts to ensure that the design of AI models directly reflects real-world requirements.

**Data augmentation.** The success of our newly introduced data augmentation technique (2×3 montage) in enhancing model robustness against the P3.0-1 OOD test dataset (a change in capturing device) demonstrates the importance of employing task-specific and domain knowledge-aware data augmentation strategies. However, this achievement is tempered by the inability of techniques to effectively address the OOD challenge presented by P3.0-2 test dataset (a change in capturing device and two more additional eggs that are not considered during fine-tuning). The morphological similarities between different helminth eggs probably affects the fine-tuned models, as well as the augmentation technique, in discerning cross-class similarities. These observations imply a dual necessity. First, a more profound integration of domain expertise is required during the development stages to ensure that the subtleties of deployment environments and contexts are thoroughly understood and represented. Second, more adaptive training strategies that consider potential OOD scenarios need to be developed. Such strategies will better prepare models for a wide range of real-world challenges, allowing for fine-tuning to specific tasks during deployment.

**Rare scenarios.** Models are limited in identifying eggs that are severely occluded, overlapped, or out-of-focus, emphasizing the crucial role of comprehensive training data. Indeed, these challenges necessitate the integration of uncommon, yet clinically vital, instances into the training examples, ensuring these scenarios are represented proportionately in comparison to more frequent ones. This could be mitigated by including examples of occluded, overlapped, and out-of-focus eggs, either by collecting additional training samples or using advanced data augmentation techniques such as selective blurring, CutMix, MixUp, and random occlusions during training. This way, models will be better prepared to mitigate false negative predictions during deployment.

## 6. Conclusions and directions for future research

Despite being labor-intensive, requiring substantial expertise, and exhibiting variable sensitivity and specificity, along with challenges such as the rapid degradation of hookworm eggs, the KK technique continues to be the gold standard for diagnosing STH and *S. mansoni* infections. Following the advent of AI and machine learning techniques in the medical sector, numerous automated processes for analyzing parasite eggs from microscopic images have been proposed, demonstrating remarkable effectiveness in controlled environments or ID datasets. However, to ensure the successful application of AI or machine learning solutions in real-world scenarios, it is crucial to evaluate model performance not only on ID data but also on OOD datasets. This type of evaluation helps guarantee that models maintain robustness and generalizability when faced with conditions and variations not seen during training.

In this study, we therefore primarily investigated the effectiveness of YOLOv7 model variants when evaluated on ID and OOD datasets containing STH and *S. mansoni* eggs from stool microscopy. Our experimentation involved correcting annotation errors found in a benchmarked ID dataset (P1.5); fine-tuning and evaluating YOLOv7 model variants using an improved version of the ID (P1.5 V2) and OOD (P3.0) datasets; testing a new data augmentation technique (montage 2×3) to increase the generalizability of models to OOD scenarios; and providing insights into the error types using TIDE, as well as visualizing activations of sample FN and FP predictions using Grad-CAM.

All variants of the YOLOv7 model demonstrated strong effectiveness when evaluated on the ID dataset. The YOLOv7-E6E variant, in particular, achieved a weighted average F1-score of 97.47%. The YOLOv7-tiny variant also performed well, especially considering its suitability for edge devices.

However, all variants showed a notable decline in performance when evaluated on OOD datasets, which included changes in the capturing device and the introduction of new egg types. In addressing OOD scenarios caused by a change in the capturing device, the newly introduced 2 × 3 montage augmentation technique improved the *AP*@*IoU*_50_ of the basic YOLOv7 model by 21.36%. Despite the significant improvement in the metrics, the performance values remain relatively low, with the *AP*@*IoU*_50_ still below 50%, necessitating further exploration of solutions to address the OOD challenge.

Our findings underscore that while predictive models demonstrate high effectiveness when evaluated using ID datasets, this effectiveness does not necessarily translate to real-world scenarios where OOD conditions can arise.

Our research has highlighted the critical need for thorough OOD analysis to ensure model reliability in real-world applications. The substantial drop in performance observed when evaluating YOLOv7 variants on OOD datasets emphasizes the importance of considering factors such as changes in imaging devices and the presence of unseen classes. By conducting a comprehensive OOD analysis, we have demonstrated the limitations of relying solely on ID performance metrics, reinforcing the necessity for developing robust models that can effectively adapt to unexpected variations and diverse environments. This focus is particularly vital in the context of deploying AI for diagnostic purposes in field settings, where variability is inevitable.

Therefore, to develop trustworthy, AI-powered, field-adaptable diagnostic devices that align with the WHO’s goal of eliminating STH and *S. mansoni* infections by 2030 as a public health problem, it is essential to rigorously evaluate AI models on OOD datasets and incorporate techniques to enhance their robustness in diverse, real-world conditions. Realizing such an AI model reduces observer-dependent variability and the labor-intensive nature of manual analysis, with broader significance that extends beyond the automation of parasitological and epidemiological research, medical diagnostics, and public health interventions. It also delivers rapid and reliable solutions, aligning with the evolving landscape of digital pathology.

As addressing OOD generalization is crucial, future work on incorporating uncertainty quantification has the potential to further improve trustworthiness by, e.g., flagging cases where predictions may be less reliable, especially in OOD scenarios where inputs deviate from the training distribution. Additionally, techniques such as OOD dataset curation and domain adaptation can be explored to enhance model reliability and improve classification performance in challenging scenarios, such as morphological similarities between eggs.

## Supporting information

S1 Fig Learning curves illustrating model performance during fine-tuning experiments (PDF)

S1 Data Spreadsheet containing the hyperparameters used for model fine-tuning. (XLSX)

S1 Text Supplementary explanation of the basic Kato-Katz (KK) procedure. (PDF)

## References

[1] World Health Organization. Ending the neglect to attain the Sustainable Development Goals: A road map for neglected tropical diseases 2021–2030. 2021. https://www.who.int/publications/i/item/9789240010352

[2] GBD 2021 Diseases and Injuries Collaborators. Global incidence, prevalence, years lived with disability (YLDs), disability-adjusted life-years (DALYs), and healthy life expectancy (HALE) for 371 diseases and injuries in 204 countries and territories and 811 subnational locations, 1990–2021: a systematic analysis for the Global Burden of Disease Study 2021. Lancet. 2024;403(10440):2133–61. doi: 10.1016/S0140-6736(24)00757-8 38642570 PMC11122111

[3] Momčilović S, Cantacessi C, Arsić-Arsenijević V, Otranto D, Tasić-Otašević S. Rapid diagnosis of parasitic diseases: current scenario and future needs. Clin Microbiol Infect. 2019;25(3):290–309. doi: 10.1016/j.cmi.2018.04.028 29730224

[4] Alva A, Cangalaya C, Quiliano M, Krebs C, Gilman RH, Sheen P, et al. Mathematical algorithm for the automatic recognition of intestinal parasites. PLoS One. 2017;12(4):e0175646. doi: 10.1371/journal.pone.0175646 28410387 PMC5391948

[5] Ward P, Broadfield LA, Dahlberg P, Leta G, Mekonnen Z, Nabatte B. The development of an artificial intelligence-based digital pathology for neglected tropical diseases: a platform specific analysis of the World Health Organization diagnostic target product profile for soil-transmitted helminthiasis. Frontiers in Tropical Diseases. 2022;3:990304. doi: 10.3389/FITD.2022.990304

[6] Genchi M, Potters I, Kaminsky RG, Montresor A, Magnino S. Bench aids for intestinal parasites. World Health Organization. 2019.

[7] Coffeng LE, Vlaminck J, Cools P, Denwood M, Albonico M, Ame SM, et al. A general framework to support cost-efficient fecal egg count methods and study design choices for large-scale STH deworming programs-monitoring of therapeutic drug efficacy as a case study. PLoS Negl Trop Dis. 2023;17(5):e0011071. doi: 10.1371/journal.pntd.0011071 37196017 PMC10228800

[8] Cools P, Vlaminck J, Albonico M, Ame S, Ayana M, José Antonio BP, et al. Diagnostic performance of a single and duplicate Kato-Katz, Mini-FLOTAC, FECPAKG2 and qPCR for the detection and quantification of soil-transmitted helminths in three endemic countries. PLoS Negl Trop Dis. 2019;13(8):e0007446. doi: 10.1371/journal.pntd.0007446 31369558 PMC6675048

[9] Saeed MA, Jabbar A. “Smart Diagnosis” of parasitic diseases by use of smartphones. J Clin Microbiol. 2017;56(1):e01469-17. doi: 10.1128/JCM.01469-17 29046408 PMC5744201

[10] Ward P, Dahlberg P, Lagatie O, Larsson J, Tynong A, Vlaminck J, et al. Affordable artificial intelligence-based digital pathology for neglected tropical diseases: a proof-of-concept for the detection of soil-transmitted helminths and Schistosoma mansoni eggs in Kato-Katz stool thick smears. PLoS Negl Trop Dis. 2022;16(6):e0010500. doi: 10.1371/journal.pntd.0010500 35714140 PMC9258839

[11] Ward PK, Roose S, Ayana M, Broadfield LA, Dahlberg P, Kabatereine N, et al. A comprehensive evaluation of an artificial intelligence based digital pathology to monitor large-scale deworming programs against soil-transmitted helminths: a study protocol. PLoS One. 2024;19(10):e0309816. doi: 10.1371/journal.pone.0309816 39466830 PMC11515989

[12] Kumar S, Arif T, Alotaibi AS, Malik MB, Manhas J. Advances towards automatic detection and classification of parasites microscopic images using deep convolutional neural network: methods, models and research directions. Arch Comput Methods Eng. 2023;30(3):2013–39. doi: 10.1007/s11831-022-09858-w 36531561 PMC9734923

[13] Butploy N, Kanarkard W, Maleewong Intapan P. Deep learning approach for ascaris lumbricoides parasite egg classification. J Parasitol Res. 2021;2021:6648038. doi: 10.1155/2021/6648038 33996149 PMC8096572

[14] Libouga IO, Bitjoka L, Gwet DLL, Boukar O, Nlôga AMN. A supervised U-Net based color image semantic segmentation for detection & classification of human intestinal parasites. e-Prime - Adv Electric Eng Electron Energy. 2022;2:100069. doi: 10.1016/j.prime.2022.100069

[15] Oyibo P, Jujjavarapu S, Meulah B, Agbana T, Braakman I, van Diepen A, et al. Schistoscope: an automated microscope with artificial intelligence for detection of schistosoma haematobium eggs in resource-limited settings. Micromachines (Basel). 2022;13(5):643. doi: 10.3390/mi13050643 35630110 PMC9146062

[16] Kitvimonrat A, Hongcharoen N, Marukatat S, Watcharabutsarakham S. Automatic detection and characterization of parasite eggs using deep learning methods. In: 2020 17th International Conference on Electrical Engineering/Electronics, Computer, Telecommunications and Information Technology (ECTI-CON). 2020. p. 153–6. 10.1109/ecti-con49241.2020.9158084

[17] Li Q, Li S, Liu X, He Z, Wang T, Xu Y, et al. FecalNet: automated detection of visible components in human feces using deep learning. Med Phys. 2020;47(9):4212–22. doi: 10.1002/mp.14352 32583463

[18] Yang A, Bakhtari N, Langdon-Embry L, Redwood E, Grandjean Lapierre S, Rakotomanga P, et al. Kankanet: an artificial neural network-based object detection smartphone application and mobile microscope as a point-of-care diagnostic aid for soil-transmitted helminthiases. PLoS Negl Trop Dis. 2019;13(8):e0007577. doi: 10.1371/journal.pntd.0007577 31381573 PMC6695198

[19] Oliveira BAS, Moreira JMP, Coelho PRS, Negrão-Corrêa DA, Geiger SM, Guimarães FG. Automated diagnosis of schistosomiasis by using faster R-CNN for egg detection in microscopy images prepared by the Kato–Katz technique. Neural Comput Appl. 2022;34(11):9025–42. doi: 10.1007/s00521-022-06924-z

[20] Ruiz-Santaquiteria J, Pedraza A, Vallez N, Velasco A. Parasitic egg detection with a deep learning ensemble. In: 2022 IEEE International Conference on Image Processing (ICIP). 2022. p. 4283–6. 10.1109/icip46576.2022.9897858

[21] Dacal E, Bermejo-Peláez D, Lin L, Álamo E, Cuadrado D, Martínez Á, et al. Mobile microscopy and telemedicine platform assisted by deep learning for the quantification of Trichuris trichiura infection. PLoS Negl Trop Dis. 2021;15(9):e0009677. doi: 10.1371/journal.pntd.0009677 34492039 PMC8448303

[22] Anantrasirichai N, Chalidabhongse TH, Palasuwan D, Naruenatthanaset K, Kobchaisawat T, Nunthanasup N, et al. ICIP 2022 challenge on parasitic egg detection and classification in microscopic images: dataset, methods and results. In: 2022 IEEE International Conference on Image Processing (ICIP). 2022. p. 4306–10. 10.1109/icip46576.2022.9897267

[23] Ragab MG, Abdulkadir SJ, Muneer A, Alqushaibi A, Sumiea EH, Qureshi R, et al. A comprehensive systematic review of YOLO for medical object detection (2018 to 2023). IEEE Access. 2024;12:57815–36. doi: 10.1109/access.2024.3386826

[24] Wang CY, Bochkovskiy A, Liao HYM. YOLOv7: trainable bag-of-freebies sets new state-of-the-art for real-time object detectors. 2023. https://github.com/WongKinYiu/yolov7

[25] Elgendy M. Deep Learning for Vision Systems; 2020. https://www.oreilly.com/library/view/deep-learning-for/9781617296192/

[26] UGent-imec. JupyterHub at imec. https://doc.ilabt.imec.be/ilabt/jupyter/index.html

[27] Bolya D, Foley S, Hays J, Hoffman J. TIDE: a general toolbox for identifying object detection errors. Lecture notes in computer science. Springer; 2020. p. 558–73. 10.1007/978-3-030-58580-8_33

[28] Selvaraju RR, Cogswell M, Das A, Vedantam R, Parikh D, Batra D. Grad-CAM: visual explanations from deep networks via gradient-based localization. Int J Comput Vis. 2019;128(2):336–59. doi: 10.1007/s11263-019-01228-7

[29] Tommy ngX. YOLOv7-GradCAM: Implementing GradCAM visualization; 2022. https://github.com/tommyngx/yolov7-GradCAM

[30] Yang J, Zhou K, Li Y, Liu Z. Generalized out-of-distribution detection: a survey. Comput Vis Pattern Recognit. 2021. doi: 10.48550/arXiv.2110.11334

[31] Lardín C. Helminths: handbook for identification and counting of parasitic helminth eggs in urban wastewater. wio. 2014;13. doi: 10.2166/9781780407159

