# Supplementary material for: Detecting soil-transmitted helminth and Schistosoma mansoni eggs in Kato-Katz stool smear microscopy images: A comprehensive in- and out-of-distribution evaluation of YOLOv7 variants
Source: PLoS Negl Trop Dis. 2025 Jul 3;19(7):e0013234. doi: 10.1371/journal.pntd.0013234 (PMC12251349; doi:10.1371/journal.pntd.0013234)
Supplement: S1 Fig — (PDF) [file pntd.0013234.s001.pdf]

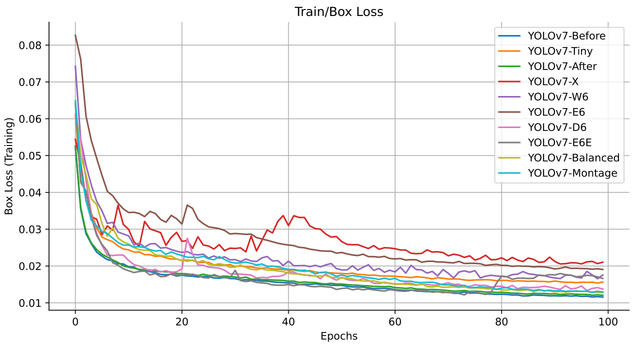

(a) Training Box Loss. The error between predicted bounding boxes and ground truth bounding boxes over 100 epochs on the training dataset.

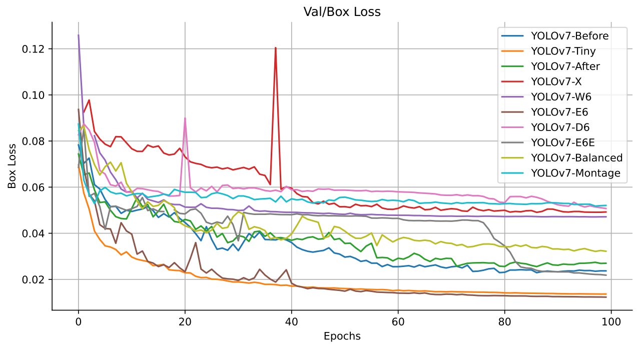

(b) Validation Box Loss. The error between predicted bounding boxes and ground truth bounding boxes over 100 epochs on the validation dataset.

Fig A. Box Loss Curves.

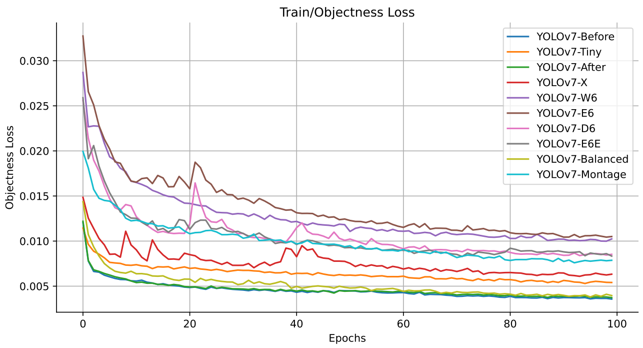

(a) Training Objectness Loss. The error in predicting the probability of object presence within proposed bounding boxes over 100 epochs on the training dataset.

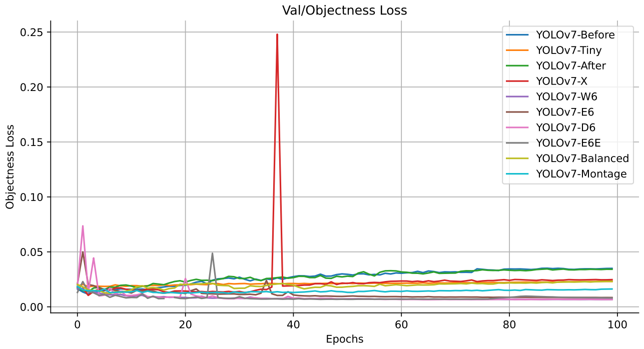

(b) Validation Objectness Loss. The error in predicting the probability of object presence within proposed bounding boxes over 100 epochs on the validation dataset.

Fig B. Objectness Loss Curves.

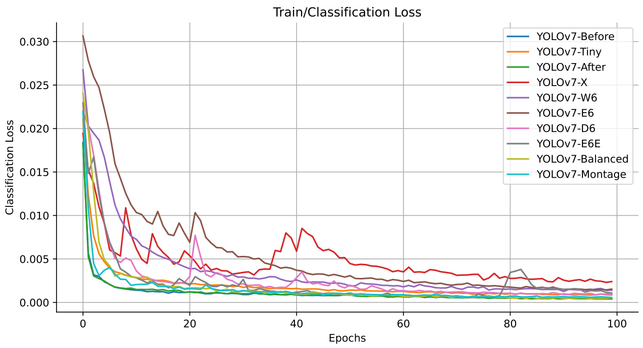

(a) Training Classification Loss. The error in predicting correct class labels for detected objects over 100 epochs on the training dataset.

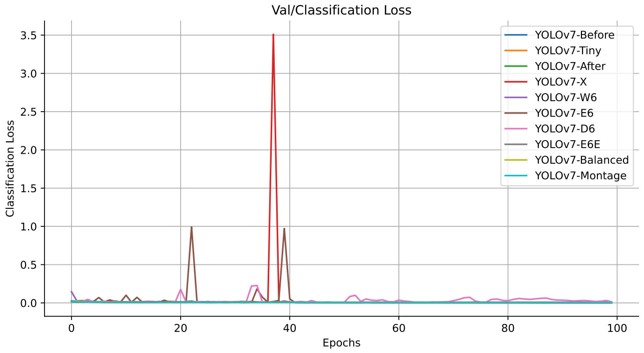

(b) Validation Classification Loss. The error in predicting correct class labels for detected objects over 100 epochs on the validation dataset.

Fig C. Classification Loss Curves.

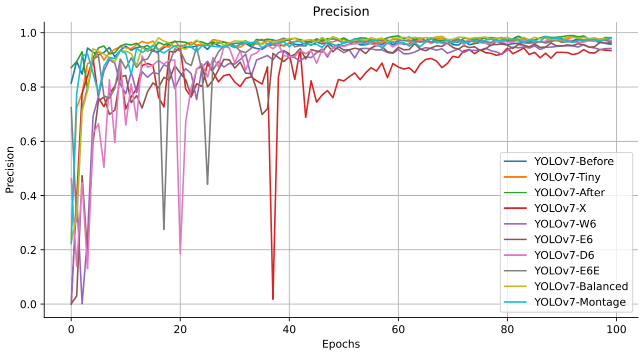

(a) Precision Curve. Precision of the models over 100 epochs.

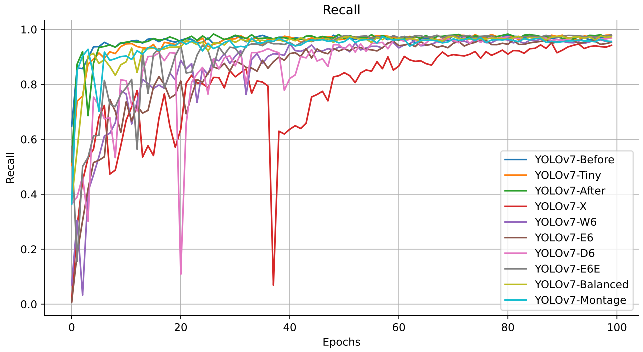

(b) Recall. Recall of the models over 100 epochs.

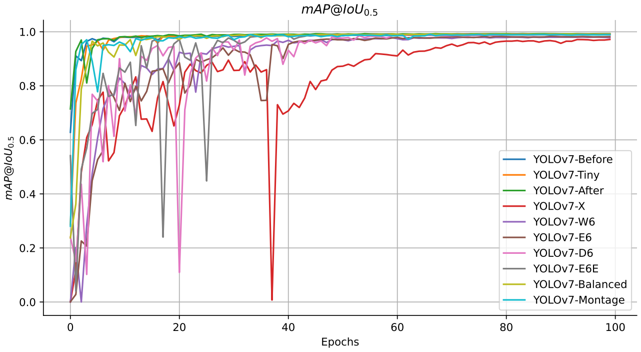

(c) $mAP@IOU_{0.5}$. $mAP@IOU_{0.5}$ of the models over 100 epochs.

Fig D. Metrics Curves.
