# Supplementary material for: Detecting soil-transmitted helminth and Schistosoma mansoni eggs in Kato-Katz stool smear microscopy images: A comprehensive in- and out-of-distribution evaluation of YOLOv7 variants
Source: PLoS Negl Trop Dis. 2025 Jul 3;19(7):e0013234. doi: 10.1371/journal.pntd.0013234 (PMC12251349; doi:10.1371/journal.pntd.0013234)
Supplement: S1 Text — (PDF) [file pntd.0013234.s003.pdf]

Kato-Katz (KK) Technique

The Kato-Katz (KK) technique is the recommended diagnostic method for monitoring large-scale treatment programs aimed at controlling soil-transmitted helminth infections. Its simple format and ease of use in field settings make it a practical and widely adopted approach for epidemiological surveys and deworming initiatives. Additionally, it provides a cost-effective means of estimating infection intensity. The procedure consists of the following key steps as discussed in [1]:

1. **Sample Collection** – A fresh stool sample is collected in a clean, dry container.
2. **Preparation of Materials** – Essential materials include a Kato-Katz template with a predefined hole size (e.g., 9 mm diameter with a thickness of 1.5 mm, delivering approximately 20 mg of stool), microscope slides, a fine-mesh nylon or plastic sieve (60–105 µm), cellophane strips pre-soaked in a glycerol-malachite green solution, a spatula, and a light microscope.
3. **Sieving the Stool** – A portion of the stool sample is pressed through a fine-mesh sieve to remove coarse debris, ensuring a uniform sample for analysis.
4. **Filling the Template** – A labeled microscope slide is placed beneath the Kato-Katz template, which is then filled with sieved stool using a spatula. Excess material is carefully leveled to ensure a standardized sample volume.
5. **Template Removal and Disinfection** – The template is gently lifted to prevent sample disruption and placed in a disinfectant solution for reuse.
6. **Covering with Cellophane** – A pre-soaked cellophane strip (glycerol-malachite green solution) is placed over the smear, facilitating clearing by rendering the fecal material transparent.
7. **Clearing the Smear** – The slide is firmly pressed against a smooth surface to spread the sample evenly. It is then left to clear at room temperature for 30–60 minutes, during which the glycerol dehydrates and clarifies the sample, allowing egg visualization.
8. **Microscopic Examination** – The cleared slide is examined under a light microscope at 10× magnification, with 40× used for detailed observations. Helminth eggs are identified based on morphological characteristics.
9. **Egg Counting and Quantification** – The number of eggs observed is multiplied by a conversion factor based on the template size to estimate the number of eggs per gram (EPG) of stool. Common multiplication factors include:
   - 50 for a 20 mg template
   - 24 for a 41.7 mg template
   - 20 for a 25 mg template
10. **Interpretation and Reporting** – Infection intensity is classified according to WHO-recommended thresholds for each parasite species, guiding treatment strategies and public health interventions.

References

1. Genchi M, Potters I, Kaminsky RG, Montresor A, Magnino S. Bench aids for intestinal parasites WHO. World Health Organization. 2019; p. 32 p.
